# Solithromycin inhibits IL-13-induced goblet cell hyperplasia and MUC5AC, CLCA1, and ANO1 in human bronchial epithelial cells

**DOI:** 10.7717/peerj.14695

**Published:** 2023-01-17

**Authors:** Yasuhiro Kimura, Masahiro Shinoda, Masaharu Shinkai, Takeshi Kaneko

**Affiliations:** 1 Department of Pulmonology, Yokohama City University Graduate School of Medicine, Yokohama, Kanagawa, Japan; 2 Department of Respiratory Medicine, Tokyo Shinagawa Hospital, Shinagawa, Tokyo, Japan

**Keywords:** Solithromycin, Goblet cell, SAM pointed domain containing ETS transcription factor, Chloride channel accessory 1, Mucin, MUC5AC, Anoctamin-1

## Abstract

Solithromycin is a novel fluoroketolide antibiotic belonging to the class of macrolide antibiotics. Activation of the interleukin (IL)-13 receptor leads to STAT6 activation and subsequent induction of SAM pointed domain containing ETS transcription factor (SPDEF), chloride channel accessory 1 (CLCA1), and anoctamin-1 (ANO1), all of which are associated with the induction of MUC5AC. We examined the effects of solithromycin on mucin production led by IL-13 signaling. Normal human bronchial epithelial cells were grown at the air-liquid interface with IL-13 with/without solithromycin for 14 days. Histochemical analysis was performed using hematoxylin and eosin staining and MUC5AC immunostaining. *MUC5AC*, *SPDEF*, *CLCA1*, and *ANO1* mRNA expressions were examined using real-time polymerase chain reaction. Western blot analysis was performed to assess CLCA1 and ANO1 proteins, and phosphorylation of STAT6 and ERK. Solithromycin attenuated IL-13 induction of goblet cell hyperplasia and *MUC5AC*, *CLCA1* and *ANO1* mRNA and protein expression induced by IL-13, but had no effect on the phosphorylation of STAT6 and ERK. Our results indicate that solithromycin could attenuate goblet cell hyperplasia and MUC5AC induced by IL-13 through inhibition of *CLCA1* and *ANO1* mRNA and protein expression. However, much more information is required to clarify the molecular mechanisms underlying the inhibition of CLCA1 and ANO1 by solithromycin.

## Introduction

Asthma is a syndrome characterized by functional and structural abnormalities, including airway hyperresponsiveness, chronic inflammation, and mucus hypersecretion. Interleukin (IL)-13, one of the cytokines produced by type 2 helper T cells (Th2), plays a major role in the pathogenesis of asthma. IL-13 induces goblet cell hyperplasia in human bronchial cells (Kuperman et al., 2002; Tanabe et al., 2008) and goblet cells produce the gel-forming mucin MUC5AC (Thornton, Rousseau & McGuckin, 2008).

Activation of IL-13 receptor leads to a signaling cascade for MUC5AC production, starting with activation of signal transducer and activator of transcription 6 (STAT6) (Kuperman et al., 2002). This is thought to be followed by the activation of SAM pointed domain containing ETS transcription factor (SPDEF) and chloride channel accessory 1 (CLCA1) (Yu et al., 2010; Alevy et al., 2012; Park et al., 2007).

Transmembrane protein (TMEM) 16A (also known as anoctamin-1 (ANO1)) is a calcium-activated chloride channel (CaCC) that belongs to a family of 10 proteins operating as phospholipid scramblases and chloride channels (Pedemonte et al., 2014). Many studies have identified TMEM16A expression and CLCA1, a regulator of CaCC, as involved in goblet cell metaplasia and mucus production/secretion (Huang et al., 2012a; Kondo et al., 2017; Benedetto et al., 2019; Miner et al., 2019; Lin et al., 2015a; Cabrita et al., 2021; Patel, Brett & Holtzman, 2009).

Epidermal growth factor receptor (EGFR) is another important modulator of mucin expression that is activated in asthma. Signaling through EGFR increases transcription of MUC5AC *via* extracellular signal-regulated kinase (ERK1/2) signaling pathways (Rose & Voynow, 2006a).

Solithromycin is a novel fluoroketolide antibiotic belonging to the class of macrolide antibiotics. Several structural modifications in solithromycin increase its binding to the ribosome and reduce its propensity for known macrolide resistance mechanisms compared to its macrolide and ketolide predecessors. Oral solithromycin has been found to be non-inferior to oral moxifloxacin in the treatment of community-acquired bacterial pneumonias, indicating the potential of macrolide monotherapy for this pathology (Barrera et al., 2016). Macrolide antibiotics have already been shown to have not only antibiotic effects, but also immunomodulatory and mucoregulatory properties (Zarogoulidis et al., 2012). Macrolide agents exert suppressive effects on mucus hypersecretion induced by several stimuli, including IL-13, both *in vivo* and *in vitro* (Tanabe et al., 2011; Kitano et al., 2011; Kaneko et al., 2003; Mertens, Hiemstra & Taube, 2016). Solithromycin has not yet been shown to exert the mucoregulatory properties seen with other macrolides. We therefore investigated the effects of solithromycin on IL-13-induced signaling pathways for mucin production using normal human bronchial epithelial (NHBE) cells.

## Materials and Methods

### Culture and differentiation of NHBE cells

NHBE cells (lot no. 0000442483; Lonza Walkersville, Walkersville, MD) were plated at 3,500 cells/cm^2^ in culture flasks of bronchial epithelial cell growth medium supplemented with the SingleQuot^^®^^ kit (BEGM medium, CC-3170; Lonza Walkersville, Walkersville, MD) and cultured at 37 °C in a 5% CO_2_ incubator. The medium was changed every 48–72 h and cells were grown to confluence for six days.

### IL-13 and solithromycin exposure

After achieving confluence, the apical medium was removed and cells were grown for 14 days at the air-liquid interface (ALI) at 37 °C in a 5% CO_2_ incubator. First, we investigated the concentrations of solithromycin that would have no effect on the differentiation of NHBE cells. When we used solithromycin at 30 µg/mL, all NHBE cells died by day 6. In addition to these results, referring to evidence regarding epithelial lining fluid in human airways (Rodvold et al., 2012) and a study examining the effects of solithromycin in a human airway epithelial cell line stimulated with *Pseudomonas aeruginosa* lipopolysaccharide to induce overexpression of MUC5AC (Kawamoto et al., 2020), we selected the concentration of solithromycin used in this study. Medium containing IL-13 (recombinant human IL-13, five ng/mL; Sigma-Aldrich, St. Louis, MO, USA) or vehicle (acetic acid) was added to the basolateral side with solithromycin (Toyama Chemical, Tokyo, Japan) at 0, 5, 10, or 15 µg/mL. Medium containing IL-13 ± solithromycin was changed every 48–72 h. Acetic acid was used as the vehicle for solithromycin.

### Histochemical and immunohistochemical analyses

Cells on porous filters were fixed in 10% neutral-buffered formalin, embedded in paraffin and cut into 4-µm slices. Paraffin-embedded tissues were deparaffinized in xylene and rehydrated in a graded alcohol series. Slides were then immersed in hematoxylin for 4 min, followed by immersion in eosin for 2 min. After thorough washing, slides were covered and observed under light microscopy.

Samples were stained for MUC5AC with the EnVision™ FLEX and the Dako Autostainer Link 48 platform. The deparaffinization, rehydration, and target-retrieval procedures were performed using the EnVision FLEX Target Retrieval solution (high pH, 1 ×) and EnVision FLEX wash buffer (1 ×). After this procedure, tissue samples were placed on the Autostainer Link 48 platform. The instrument performed the staining process by applying the appropriate reagent, monitoring incubation time and rinsing slides between reagents. Slides were then observed under light microscopy.

### Real-time quantitative polymerase chain reaction (RT-PCR)

To examine the expressions of *MUC5AC*, *SPDEF*, *CLCA1* and *ANO1* mRNAs, total RNA was extracted from cells exposed to solithromycin or vehicle for 14 days and RT-PCR was conducted as previously described (Kitano et al., 2011). The mRNA expression of the housekeeping gene glyceraldehyde-3-phosphate dehydrogenase (GAPDH) was used as a normalization control. Primer pairs for MUC5AC were as follows: forward, 5′-TACTCCACAGACTGCACCAACTG-3′; and reverse, 5′-CGTGTATTGCTTCCCGTCAA-3′. SPDEF/CLCA1/ANO1/GAPDH primers were purchased from Takara Bio (Shiga, Japan) using the perfect Real Time Support System. Takara Primer set IDs were: SPDEF, HA191804; CLCA1, HA206922; ANO1, HA187972; and GAPDH, HA067812.

### Western blot analysis

Western blot analysis was used to examine CLCA1 and ANO1 proteins. These proteins were exposed to IL-13 (0 or five ng/mL) with solithromycin (0, 5, 10, or 15 µg/mL) for 14 days. Western blot analysis was used to examine the phosphorylation of STAT6 and ERK1/2. NHBE cells that had reached confluence were exposed to IL-13 (0 or five ng/mL) with solithromycin (0, 5, 10, or 15 µg/mL). Cell lysates were harvested after 30 min of IL-13 exposure. Protein concentration and the procedure of Western blot analysis were conducted as described previously (Kitano et al., 2011). GAPDH protein was used to normalize levels of total tissue lysate on the same membrane.

### Statistical methods

All studies were performed as duplicate trials on at least three separate occasions. Data are expressed as mean ± standard error of the mean (SEM). The Kolmogorov–Smirnov test was used to test for normality. Student’s *t*-test (two-tailed, unpaired) and the nonparametric Mann–Whitney test (two-tailed, unpaired) were used for comparisons of statistical differences between two groups. Values of *P* < 0.05 were considered significant. Data were analyzed using EZR version 1.41 (Saitama Medical Center, Jichi Medical University, Saitama, Japan).

## Results

### Effects of solithromycin on IL-13-induced goblet cell hyperplasia

**Figure 1:**
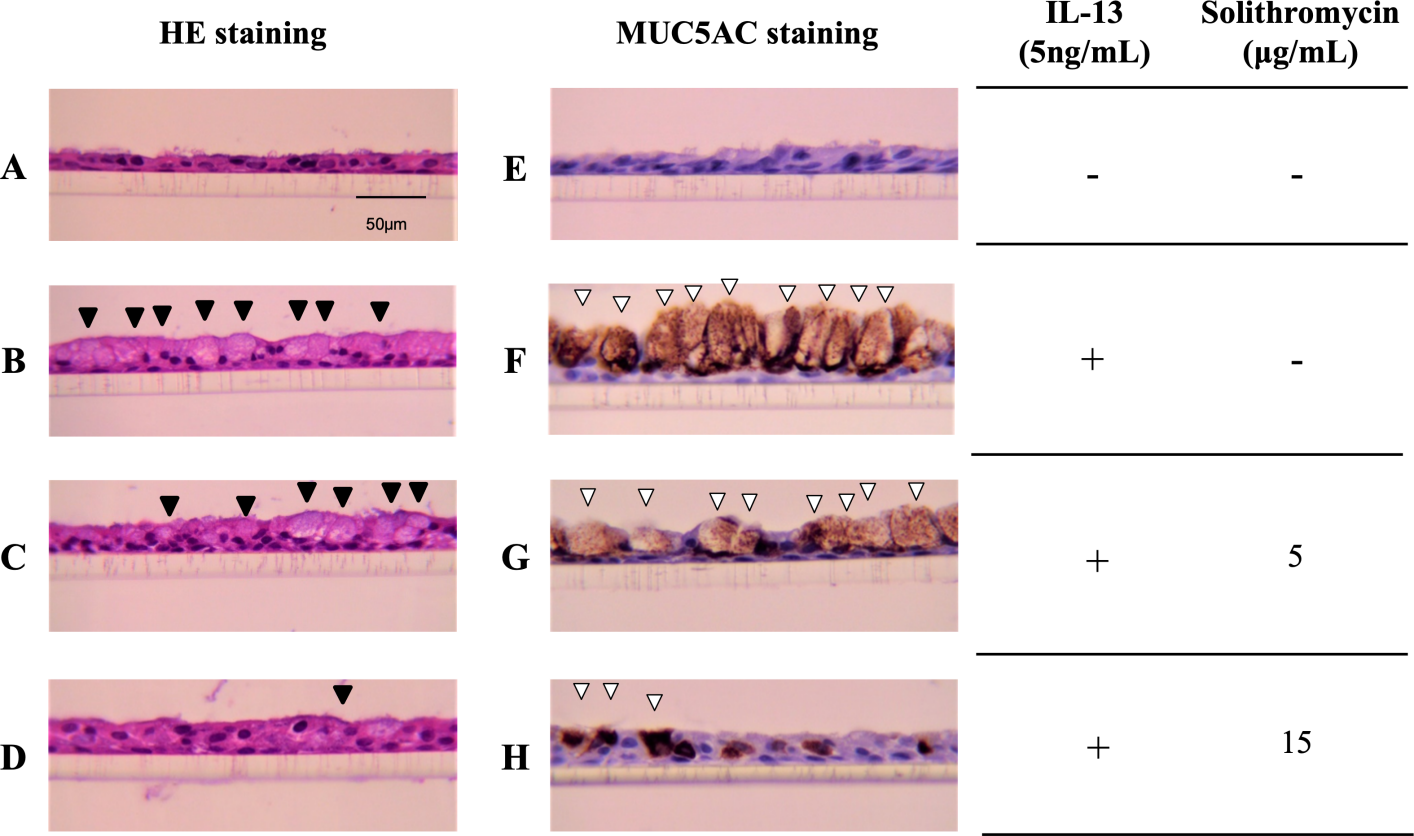
Hematoxylin and eosin and MUC5AC immunostaining of NHBE cells grown for 14 days at the air-liquid interface with IL-13 (five ng/mL) and either solithromycin. Hematoxylin and eosin (A–D) and MUC5AC immunostaining (E–H) of NHBE cells grown for 14 days at the air-liquid interface with IL-13 (5 ng/mL) (B–D, F–H) and either solithromycin vehicle (acetic acid) (A, B, E, F), solithromycin (C, G: 5 µg/mL, D, H: 15 µg/mL) or IL-13 vehicle (PBS) (A, E). Black arrowheads show goblet cells with secretory granules. White arrowheads show MUC5AC-positive cells.

NHBE cells cultured at the ALI for 14 days demonstrated a well-differentiated morphology with ciliated cells at the surface of epithelial layers (Fig. 1A). In the presence of IL-13, NHBE cells differentiated into goblet cells showing secretory granules (Fig. 1B) and showed increased immunostaining for MUC5AC (Fig. 1F). Solithromycin administered concomitantly decreased the number of goblet cells (Figs. 1C, 1D) and MUC5AC-positive cells compared with IL-13 and solithromycin vehicle (Figs. 1G, 1H).

### Effects of solithromycin on IL-13-stimulated *MUC5AC* gene expression

**Figure 2:**
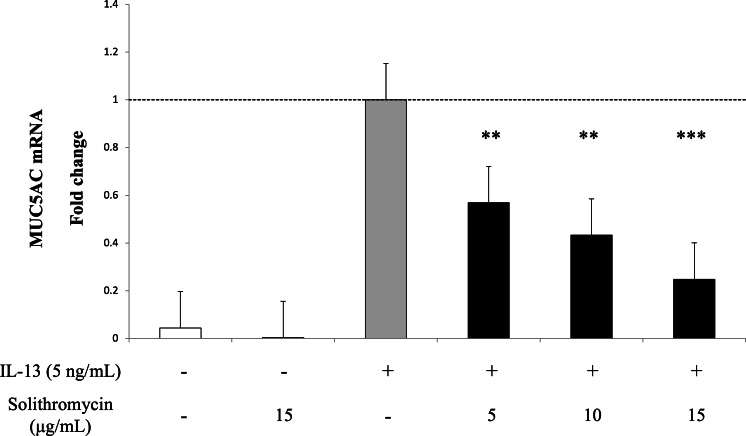
*MUC5AC* mRNA expression in NHBE cells grown for 14 days with basal exposure to IL-13 (0 or 5 ng/mL) and solithromycin (0, 5, 10 or 15 µg/mL). *MUC5AC* mRNA was measured by real-time PCR. Data are shown as mean ± SEM from samples performed three times separately. Solithromycin decreases *MUC5AC* mRNA in a dose-dependent manner. Significant differences from IL-13 alone are indicated by ** *p* < 0.01, *** *p* < 0.001.

Exposure to IL-13 over 14 days increased *MUC5AC* mRNA expression. While adding solithromycin, a dose-dependent decrease in *MUC5AC* mRNA was identified (Fig. 2).

### Effects of solithromycin on *SPDEF* mRNA, *CLCA1* and *ANO1* mRNA and protein expression

**Figure 3:**
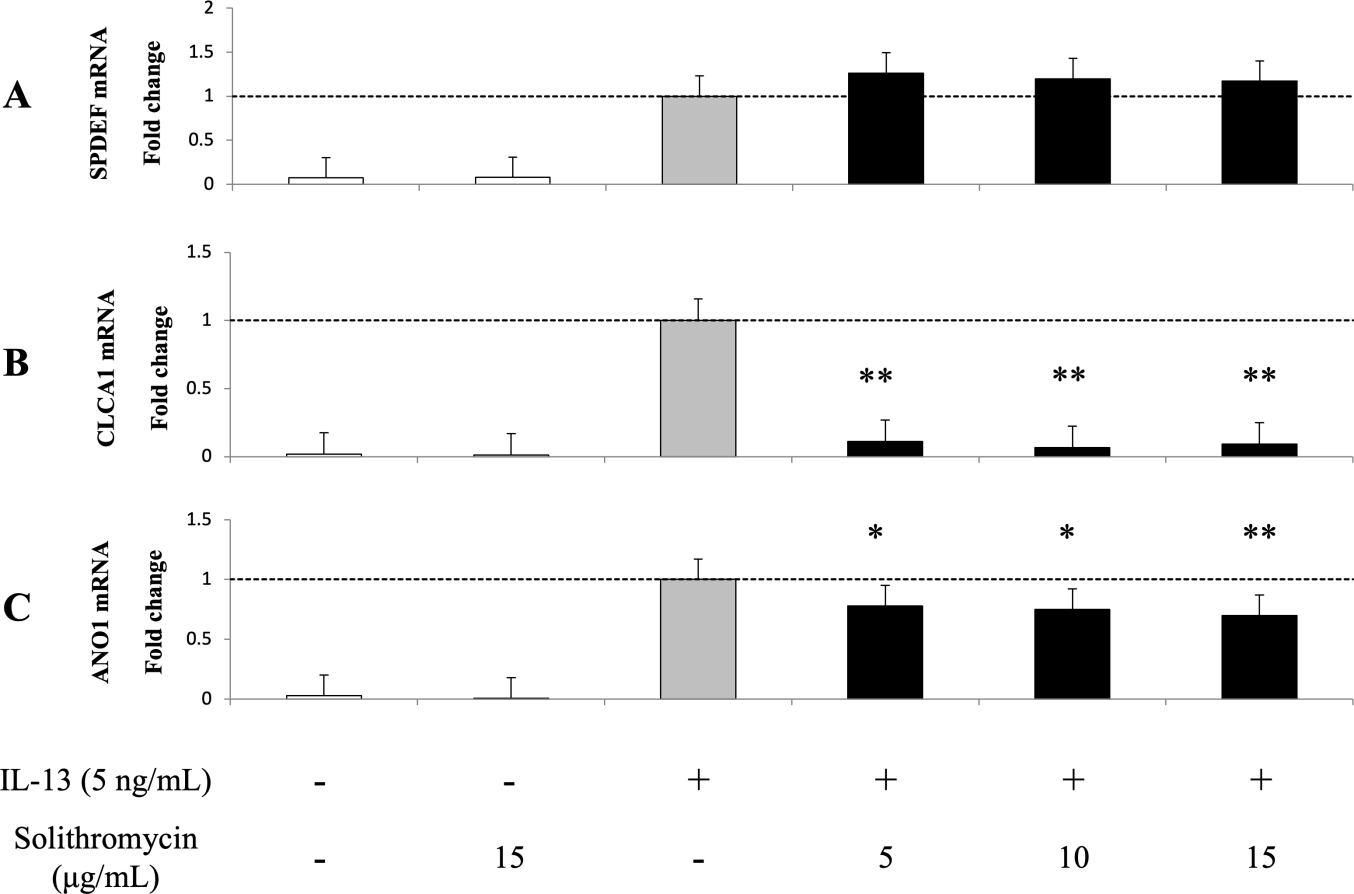
Expression of *SPDEF*, *CLCA1*, and *ANO1* in NHBE cells grown for 14 days with basal exposure to IL-13 (0 or 5 ng/mL) and solithromycin (0, 5, 10, or 15 µg/mL) or acetic acid. *SPDEF*, *CLCA1* and *ANO1* mRNA are measured by real-time PCR. Data are shown as mean ± SEM from samples performed three times separately. Experiments are performed three times each. Significant differences from IL-13 alone are indicated by * *p* < 0.05 and ** *p* < 0.01.

**Figure 4:**
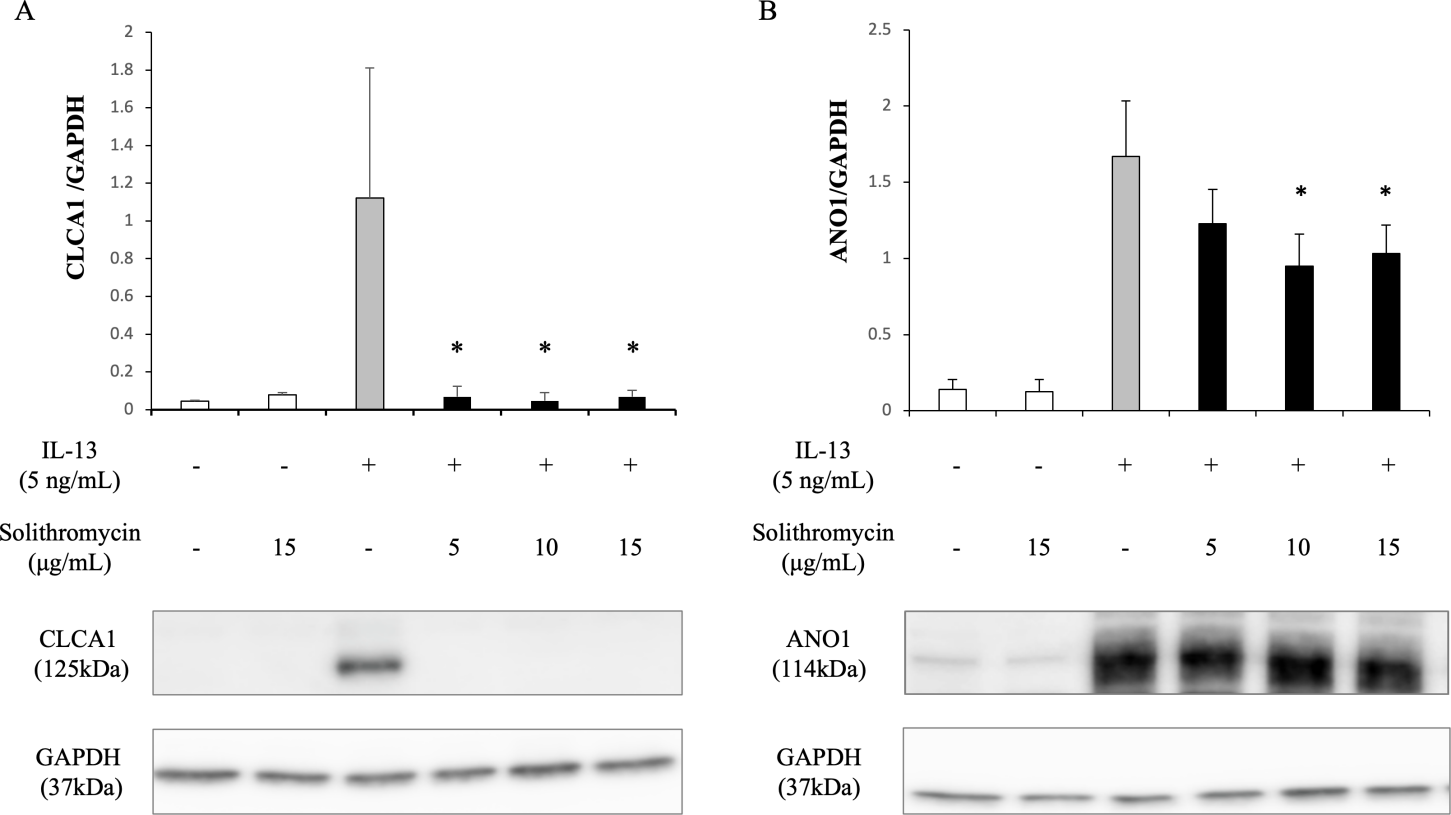
Western blot analysis of CLCA1 and ANO1 activation with exposure to IL-13 (0 or five ng/mL) and solithromycin (0, 5, 10 or 15 µg/mL) for 14 days. Data are shown as mean ± SEM from samples performed three times separately. Significant differences from IL-13 alone are indicated by an asterisk (*) *p* < 0.05.

Expression of *SPDEF* mRNA, *CLCA1* and *ANO1* mRNA and protein increased with exposure to IL-13 over 14 days compared with the IL-13 vehicle. Addition of solithromycin had no effect on *SPDEF* mRNA expression (Fig. 3A), but decreased *CLCA1* and *ANO1* mRNA and protein expression (Figs. 3B, 3C, 4A, 4B).

### Effects of solithromycin on phosphorylation of STAT6 and ERK1/2

**Figure 5:**
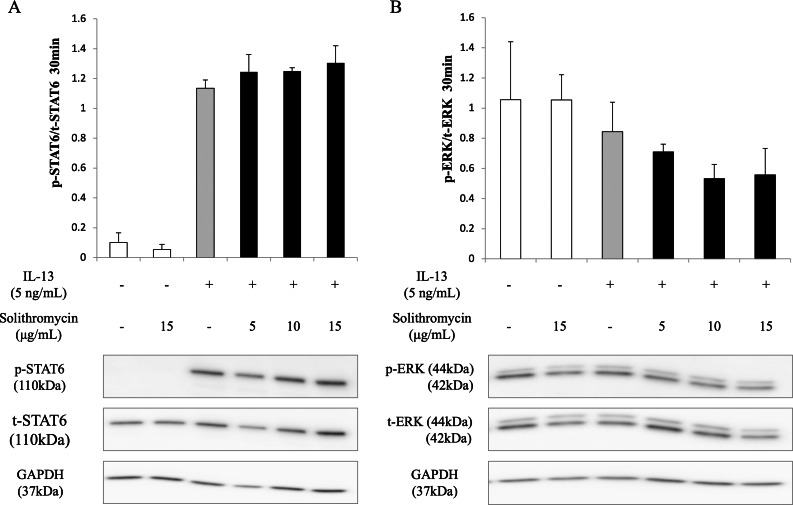
Western blot analysis of STAT6 and ERK activation with exposure to IL-13 (0 or 5 ng/mL) and solithromycin (0, 5, 10, or 15 µg/mL). Data are shown as means ± SEM of the ratio of phosphorylated (p)-STAT6/total (t)-STAT6 from samples performed three times separately. NHBE cells were harvested after 30 min of exposure to IL-13 and solithromycin. While IL-13 increased the ratio of p-STAT6/t-STAT6, solithromycin did not affect on. IL-13 did not increase the ratio of p-ERK/t-ERK, and solithromycin did not affect the ratio of p-ERK/t-ERK.

Western blot analysis of NHBE cell lysate showed that IL-13 increased phosphorylation of STAT6 by 30 min after IL-13 stimulation, while solithromycin had no effect on STAT6 phosphorylation (Fig. 5A). IL-13 did not increase phosphorylation of ERK at 30 min and solithromycin had no effect on ERK phosphorylation (Fig. 5B).

## Discussion

This study demonstrated that solithromycin inhibited the goblet cell hyperplasia and MUC5AC expression induced by IL-13 and affected IL-13-exposed cells in attenuating CLCA1 and ANO-1 (TMEM16A), but exerted no effects on STAT6 activation, SPDEF expression or phosphorylation of ERK1/2.

MAC5AC expression by IL-13 is thought to occur through the suppression of FOXA2 by JAK1/STAT6-mediated enhancement of SPDEF expression (Zhen et al., 2007) and activation of MAPK13 by the enhancement of TMEM16A and CLCA1 expression by SPDEF (Yu et al., 2010; Alevy et al., 2012; Qin et al., 2016).

IL-13 reportedly does not induce EGFR ligand expression in airway epithelial cells *in vitro* (Zhen et al., 2007), but instead causes inflammation, increases expression of EGFR ligands, and promotes the production of MUC5AC *in vivo* (Shim et al., 2001). FOXA2 (Zhen et al., 2007), TMEM16A (Qin et al., 2016; Crottès et al., 2019), and CLCA1 (Yu et al., 2010; Nagashima et al., 2016), common to both IL-13 and EGFR pathways, are thought to act as regulatory factors for MUC5AC expression.

The function of CLCA1 is associated with mucus production and the activation of CaCCs through TMEM16A. CLCA1 engages TMEM16A directly on the cell surface, and this effect appears to stabilize the expression of TMEM16A (Yurtsever et al., 2012; Sala-Rabanal et al., 2015). Like CLCA1 expression, IL-4/IL-13 induces the expression of TMEM16A (Scudieri et al., 2012; Lin et al., 2015b). Moreover, inhibitors of TMEM16A have been reported to inhibit mucus production (Huang et al., 2012b; Zhang et al., 2015). The CLCA1-TMEM16A interaction is hypothesized to activate intracellular MAPK13 and induce MUC5AC, leading to the formation and degranulation of secretory granules.

Our results imply that solithromycin may suppress IL-13-induced goblet cell hyperplasia through the TMEM16A pathway, but not through the IL-13R *α*1-JAK-STAT6 or EGFR-ERK1/2 pathways. Solithromycin suppressed MUC5AC expression like other macrolide antibiotics. Clarithromycin reportedly suppressed IL-13-induced goblet cell hyperplasia through a TMEM16A-dependent pathway (Hara et al., 2019) and azithromycin decreased IL-13-induced MUC5AC expression by decreasing CLCA1 (Mertens et al., 2016). Solithromycin is speculated to have a similar effect. Our previous report showed that clarithromycin may inhibit IL-13-induced goblet cell hyperplasia by suppressing not only CLCA1, but also SPDEF and ERK1/2 phosphorylation (Nagashima et al., 2016). However, solithromycin does not appear to have that effect. Much more information is required to elucidate the molecular mechanisms by which solithromycin inhibits CLCA1 and TMEM16A.

As a limitation of this study, we did not measure FOXA2, a standard regulator of the IL-13 and EGFR pathways, or MAPK13, which CLCA1 activates, so we cannot fully speculate on the pathways by which solithromycin inhibits goblet cell hyperplasia. We also consider it essential to examine the effects of solithromycin on FOXA3 (Rose & Voynow, 2006b; Schroeder et al., 2012; Williams et al., 2006), which directly binds to AGR2 and MUC5AC and causes mucus production/goblet cell hyperplasia in the airway. These will be the subjects of our future research.

Solithromycin has been reported to exert potent *in vitro* activity against most common community-acquired bacterial pneumonia pathogens, including macrolide-, penicillin-, and fluoroquinolone-resistant *Streptococcus pneumonia*, *Haemophilus influenzae*, and atypical bacteria, making this an essential option as an antibacterial agent for community-acquired pneumonia (Kato et al., 2019). Solithromycin has also been known to have inhibitory effects on tumor necrosis factor *α* production from monocytic cell lines, suppresses mucin overexpression induced by *P. aeruginosa* LPS in airway epithelial cells (Kawamoto et al., 2020) and may be able to control chronic lower respiratory tract disease. Based on the present results, solithromycin may represent a useful treatment option by reducing excessive mucus secretion in asthmatic patients suffering from pneumonia and providing an additional therapeutic agent for patients with bronchial asthma with treatment-resistant sputum.

## Conclusion

We have demonstrated that solithromycin could attenuate goblet cell hyperplasia and MUC5AC induced by IL-13 through the inhibition of *CLCA1* and *TMEM16A* mRNA and protein expression.

##  Supplemental Information

10.7717/peerj.14695/supp-1 Supplemental Information 1 Fig. 2 raw data

10.7717/peerj.14695/supp-2 Supplemental Information 2 Fig. 3 raw data

10.7717/peerj.14695/supp-3 Supplemental Information 3 Fig. 4 raw data

10.7717/peerj.14695/supp-4 Supplemental Information 4 Fig. 5 raw data

## Additional Information and Declarations

### Data Availability

The following information was supplied regarding data availability: The raw measurements are available in the Supplemental Files.
